# Epidemiology of small intestine cancer in Iran

**DOI:** 10.1002/cnr2.1593

**Published:** 2021-11-25

**Authors:** Mehdi Azizmohammad Looha, Mohammad Esmaeil Akbari, Elaheh Zarean, Soheila Khodakarim

**Affiliations:** ^1^ Cancer Research Center Shahid Beheshti University of Medical Sciences Tehran Iran; ^2^ Modeling in Health Research Center Shahrekord University of Medical Sciences Shahrekord Iran; ^3^ Department of Biostatistics, School of Medicine Shiraz University of Medical Sciences Shiraz Iran

**Keywords:** cancer, epidemiology, small intestine, survival

## Abstract

**Background:**

Little is known about the epidemiology of small intestine (SI) cancer in Iran, a rare cancer entity worldwide.

**Aims:**

The aim of the present study was to investigate the incidence patterns and survival rates of SI cancer in Iran through a population‐based study.

**Methods and Results:**

Data on all reported cases of SI cancer were extracted from the Iran National Cancer Registry based on ICD‐O‐3 codes. Age‐standardized incidence rates (ASIR), age‐specific incidence rates, standardized rate ratios (SRR), time trends, and absolute survival rates were calculated.

During 2005–2015, a total of 4928 SI cancers (ASIR: 0.87/100 000) were diagnosed, including 2835 carcinomas (ASIR: 0.51), 214 neuroendocrine malignancies (ASIR: 0.04), 228 sarcomas (ASIR: 0.04), and 704 lymphomas (ASIR: 0.11). Carcinomas and lymphomas occurred more frequently in men than in women (SRR: 1.37/100 000 and 1.85/100 000, respectively), while the other two histological subtypes were almost equally distributed. 78% of carcinomas and 53% of neuroendocrine tumors were located in the duodenum. Sarcomas occurred most frequently in the jejunum (41%), while lymphomas were most frequently in the ileum (44%). From 2005 to 2015, the number of reported cases of SI cancer increased by 9.6% per year. The median age of diagnosis for women and men was 61. The absolute 5‐year survival rate was 35.3%, varying by sex, age, and subtype. Carcinomas had the lowest survival rate (24.1%) while neuroendocrine carcinomas had the highest survival rate (69.7%).

**Conclusion:**

Epidemiological patterns of SI cancer in Iran differed slightly from patterns in the United States and the United Kingdom. In contrast to other countries, the neuroendocrine form is presented as the rarest subtype in Iran. The overall incidence of SI cancer was lower in Iran than in high‐income countries. In contrast, the average prognosis of SI cancer was worse in Iran, indicating the need to improve early detection, diagnosis, and treatment.

## 1 INTRODUCTION

Small intestine (SI) cancers are recognized as the rare types of worldwide malignancies. The four main histological subtypes of SI cancers include adenocarcinomas, neuroendocrine tumors, sarcomas, and lymphomas.^1^ It is estimated that the incidence rate of SI cancer would experience a considerable growth in high‐income countries such as the United States, the United Kingdom, and France. SI consists of three different areas including the duodenum, jejunum, and ileum. One of the most common sites for tumor growth is the duodenum where a large amount of digestive enzymes of a human body is released. In previous studies, it is reported that from 55% to 88% of SI cancers occur in this smallest area of SI while jejunum figures 11%–25% of all these kinds of cancer and between 7% and 17% of the cases belongs to the ileum.^2^, ^3^, ^4^

According to recent epidemiological studies, the incidence rate of SI cancer was 2.3 per 100 000 person‐years in the United States in 2018. In addition, SI cancer was placed at 23rd in the ranking of most common cancers in this country.^1^ Researchers demonstrated that the incidence rate of SI among men was about 2.6 while this rate was 2.0 among women. Although the incidence rate of this neoplasm has gradually increased, the mortality rate has remained unchanged. Despite the fact that, deaths from SI cancer have increased in the United Kingdom, its rate of increase is less than the rate of increase in the incidence of this cancer, indicating an increase in survivals.^1^, ^5^

The 5‐year survival of SI cancer varies among various countries across the world. Although this rate was 67.6% in the United States, the average 5‐year survival of SI patients in European countries was considerably lower than that of the United States. The histology type of tumor has a pivotal role in the 5‐year survival of SI neoplasm. As the results of recent researches indicated, neuroendocrine cancers and carcinomas had the highest and lowest survival rate respectively. Several risk factors have been identified for SI cancer, including race and ethnicity, age, sex, stage, subsite, diet, alcohol, and obesity.^1^, ^6^

A few studies have been conducted to evaluate the incidence, risk factors, and survival analysis of this fatal worldwide malignancy. However, to the best of the authors' knowledge, there is no comprehensive study investigating the epidemiology of SI cancer in Iran. The aim of the present study was to conduct a population‐based study to determine the incidence pattern of SI cancer in Iran to enable health authorities, clinical experts, and policymakers to provide more targeted and earlier diagnosis and better care.

## 2 METHODS

### 2.1 Study population

For the current study data on patients with SI neoplasms were analyzed, which had been reported to the Iran National Cancer Registry (INCR), between March 20, 2005 and March 20, 2015. This is a national, mandatory registration program to collect information on clinical procedures, pathological examinations, and causes of death on cancer cases in the population. All health centers including hospitals, clinics, and pathology laboratories were required to report patient information to the INCR. Being a time interval between cancer data collection and recording these data in patient's medical records made it is not possible for us to use the updated data during the course of the study. For that reason, the latest available national data were used in this study.

The data used potentially faced with some problems; problems such as multiple recorded data for some patients. In addition, incorrect morphology may have been recorded for a topography related to SI cancer. Moreover, the diagnostic method of SI cancer may have been incorrect or invalid. Furthermore, the patients' age and date of birth did not match in some cases. Therefore, the quality of data needs to be evaluated.

The extraction and clean‐up of the analyzed dataset by the INCR was done in several steps. In the first step, the consistency of the topographical and morphological information of the patients were evaluated. If there was any inconsistency, the data would be re‐examined and if the data was inaccurate, the subject would be omitted from the study. Subsequently, the type of patients' tumor was assessed to see whether it was diagnosed based on the correct procedure used for SI or not. In the next step, the accuracy of patients' birthdays and the date of cancer diagnosis were checked; the incorrect information was modified or removed from the dataset. The pathology (or cytology), clinical outcome, and death certificate only were applied to identify the cancer diagnosis. Duplicate cases were identified through checking their first name and surname, sex, and father's name. Patients with the exactly matched records were assigned to duplicate records and were removed automatically.

### 2.2 Data variables

In the current study, patients' information regarding the topography and histology of SI was classified based on the third edition (first revision) of the International Classification of Diseases for Oncology (ICD‐O3). All types of the SI cancers (ICD‐O topography codes C17‐0‐C17.9), with malignant behavior (ICD‐O behavior code/3) were presented in four various categories including carcinomas, neuroendocrine cancers, sarcomas, and lymphomas.^7^ Other information such as sex, age, birth date, city of residence, phone number, and date of diagnosis were also registered during the data collection used in this study. Through national census data performed by the Statistical Center of Iran, the incidence rate of this cancer in different age groups was calculated while using Iran's total population in 2006, 2011, and 2016. Not having access to information regarding the population of other years caused these figures to be estimated through growth rate between two consecutive censuses. Therefore, each year's population was calculated by multiplying the population of the previous year in the growth rate.

Twenty percent of the patients whose medical record were registered in national census data were randomly selected from all over the country and were interviewed by telephone. In this telephone interview, the type of cancer recorded in the data was checked and verified. Besides, their survival status, death status due to SIs, and patient's death date were questioned and recorded.

### 2.3 Statistical analysis

Crude incidence rates (per 100 000 person‐years) and 95% confidence intervals (CIs) were calculated for total cases and each age group (age‐specific incidence rate) as follows:

ai±zα2.SEai,

in which

$$a_{i}=\text{crude incidence rate}=\frac{\text{number of}\;\mathrm{new}\;\text{cases of disease}}{\text{population}\;\mathrm{at}\;\text{risk}}\text{in}\;a\;\text{period of time},$$

$$\mathrm{SE}\left(a_{i}\right)=\sqrt{100\;000^{2}.\frac{1}{n}.\frac{r}{n}.\left(1-\frac{r}{n}\right)},$$

$Z_{\alpha /2}$ is a standardized normal deviate, *r* refers to the numbered of cases occurred in the *i*th age class and n refers to the person‐years of observation in the age class during the same period of time as cases were counted. The age‐standardized incidence rates (ASIRs) per 100 000 and 95% CI were computed based on new World Health Organization (WHO) standard population (2000–2025) in which these estimations are regarded as the weights in the standardization method. These formulas can be expressed as^8^, ^9^:

ASIR±Zα2.SEASIR,

in which

$$\text{ASIR}=\frac{\sum_{i=1}^{A}a_{i}w_{i}}{\sum_{i=1}^{A}w_{i}},$$

and

$$\mathrm{SE}\left(\text{ASIR}\right)=\sqrt{\frac{\sum_{i=1}^{A}\left[\frac{a_{i}w_{i}^{2}\left(100\;000-a_{i}\right)}{n_{i}}\right]}{\left(\sum_{I=1}^{A}w_{i}\right)}},$$

where *Z*_α/2_ is a standardized normal deviate, *a*_*i*_ is the crude incidence rate in each age category (age‐specific incidence rate), and *w*_*i*_ is the new WHO standard population. By applying a direct method, the 95% CI standardized rate ratio ($\mathrm{SRR}=\frac{\mathrm{ASI}R_{1}}{\mathrm{ASI}R_{2}}$) were obtained^10^ as follows:

$${\left(\mathrm{SRR}\right)}^{1\pm \left(\frac{Z_{\frac{\alpha }{2}}}{x}\right)},$$

in which

$$X=\frac{\mathrm{ASI}R_{1}-\mathrm{AS}R_{2}}{\sqrt{s.e.{\left(\mathrm{ASI}R_{1}\right)}^{2}+s.e.{\left(\mathrm{ASI}R_{2}\right)}^{2}}}.$$

The average annual percent change (AAPC) and 95% CI were calculated by applying Joinpoint software to summarize the ASIR trend in the study.^11^ The ASIRs, SRRs, and AAPCs were assessed according to patients' histology information and various subsites such as duodenum (C17.0), jejunum (C17.1), ileum (C17.2), Meckel's diverticulum (C17.3), overlapping sites (C17.8), and unspecified site (C17.9).

Survival information of patients with malignant behavior included all samples recorded by telephone interview, from 2005 to 2015 and patients were followed up till 2020. The 1‐ and 5‐year survival rate were computed through employing the Kaplan–Meier approach by sex, age, calendar year, and cancer's subsites. The log‐rank test was used to compare survival distribution among variable levels.^12^ The overall survival rate with 95% CI was obtained by Kaplan–Meier survival curve.^13^ The cause‐specific survival method as an alternative to relative‐survival methods was applied for estimating cancer survival, since the suitable life‐tables were not available.^14^, ^15^ The current study was approved by the Ethics Committee of Shahid Beheshti University of Medical Sciences (IR.SBMU.CRC.REC.1399.004).

## 3 RESULTS

**FIGURE 1:**
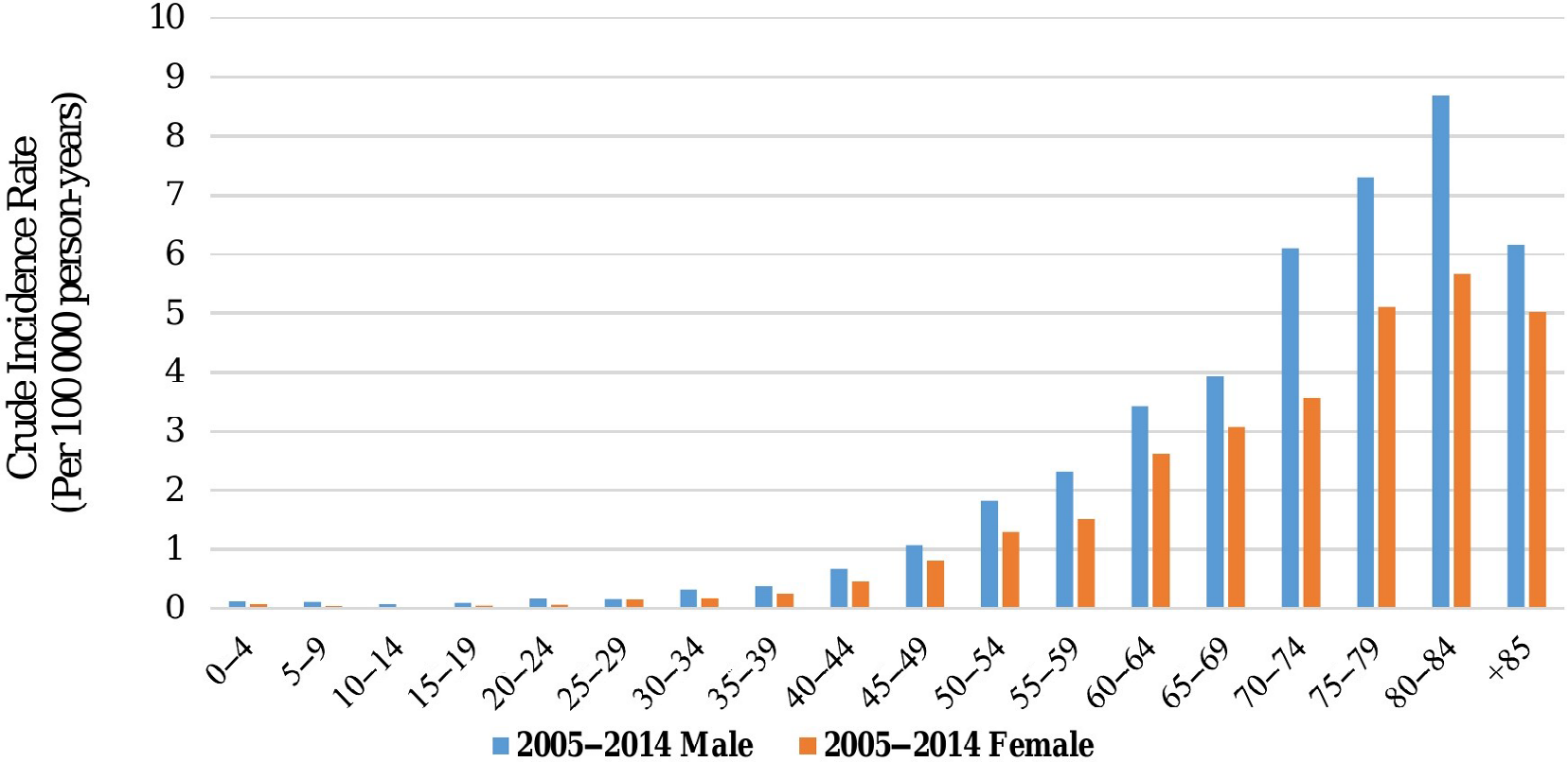
The age‐specific incidence rate (per 100 000 person‐year) of small intestine cancer, Iran, 2005–2015

In total, 4928 SI cancers were diagnosed during 2005–2015, after removing patients with wrong histology information (*n* = 80) and duplicated data (*n* = 128) (Table S1). From March 20, 2005 to March 20, 2010 and from March 21, 2010 to March 20, 2015, 1826 and 3102 cases of SI cancer had been reported, respectively. The highest frequency of SI cancers was observed in the age group of 50–80. The overall age‐specific incidence rate was higher than 1.0 per 100 000 person‐years in patients over 50 and the highest age‐specific incidence rate was observed in age group of 80–84 years with a value of 7.21 (6.47–7.95) per 100 000 person‐years. In the current study, the overall ASIR (95% CI) was 0.90 (0.85–0.90) per 100 000 person‐years, while the ASIR in males was 1.03 (0.99–1.07) and in females was 0.71 (0.68–0.74) per 100 000 person‐years (Table 1 and Table S2, Figure 1).

**TABLE 1:** The 5‐year annual frequency (age‐specific incidence rate per 100 000 person‐year) of small intestine cancer, Iran, 2005–2015

Age group	2005–2010			2010–2015			2005–2015		
	Total	Male	Female	Total	Male	Female	Total	Male	Female
0–4	17 (0.06)	12 (0.08)	5 (0.04)	40 (0.13)	24 (0.15)	16 (0.10)	57 (0.09)	36 (0.12)	21 (0.07)
5–9	25 (0.09)	18 (0.13)	7 (0.05)	17 (0.06)	13 (0.09)	4 (0.03)	42 (0.07)	31 (0.11)	11 (0.04)
10–14	15 (0.05)	10 (0.06)	5 (0.03)	13 (0.05)	11 (0.08)	2 (0.01)	28 (0.05)	21 (0.07)	7 (0.02) 0
15–19	27 (0.07)	19 (0.09)	8 (0.04)	23 (0.07)	15 (0.09)	8 (0.05)	50 (0.07)	34 (0.09)	16 (0.04)
20–24	38 (0.09)	27 (0.12)	11 (0.05)	54 (0.14)	39 (0.20)	15 (0.08)	92 (0.11)	66 (0.16)	26 (0.06)
25–29	65 (0.17)	32 (0.17)	33 (0.18)	52 (0.12)	28 (0.13)	24 (0.11)	117 (0.15)	60 (0.15)	57 (0.14)
30–34	71 (0.24)	44 (0.30)	27 (0.19)	91 (0.25)	62 (0.34)	29 (0.16)	162 (0.25)	106 (0.32)	56 (0.17)
35–39	77 (0.31)	47 (0.36)	30 (0.24)	96 (0.33)	58 (0.39)	38 (0.26)	173 (0.32)	105 (0.38)	68 (0.25)
40–44	97 (0.46)	58 (0.54)	39 (0.37)	164 (0.65)	100 (0.79)	64 (0.52)	261 (0.56)	158 (0.67)	103 (0.45)
45–49	145 (0.80)	84 (0.91)	61 (0.68)	223 (1.06)	127 (1.20)	96 (0.92)	368 (0.94)	211 (1.07)	157 (0.81)
50–54	191 (1.32)	122 (1.67)	69 (0.96)	316 (1.76)	176 (1.96)	140 (1.57)	507 (1.56)	298 (1.83)	209 (1.29)
55–59	165 (1.62)	95 (1.90)	70 (1.35)	297 (2.13)	182 (2.63)	115 (1.63)	462 (1.91)	277 (2.32)	185 (1.51)
60–64	202 (2.62)	100 (2.65)	102 (2.60)	329 (3.30)	193 (4.04)	136 (2.62)	531 (3.00)	293 (3.42)	238 (2.61)
65–69	163 (2.66)	99 (3.16)	64 (2.14)	300 (4.22)	158 (4.65)	142 (3.84)	463 (3.50)	257 (3.93)	206 (3.08)
70–74	209 (3.73)	140 (4.74)	69 (2.61)	340 (6.00)	213 (7.54)	127 (4.47)	549 (4.87)	353 (6.11)	196 (3.57)
75–79	175 (4.76)	111 (5.66)	64 (3.73)	337 (7.51)	203 (8.70)	134 (6.22)	512 (6.27)	314 (7.31)	198 (5.12)
80–84	108 (5.09)	73 (6.69)	35 (3.40)	259 (8.72)	153 (10.14	106 (7.26)	367 (7.21)	226 (8.69)	141 (5.66)
85+	36 (2.57)	17 (2.34)	19 (2.82)	151 (7.80)	89 (8.93)	62 (6.60)	187 (5.61)	106 (6.16)	81 (5.02)
Total	1826 (0.70, 0.67–0.73)^a^	1108 (0.83, 0.78–0.88)	718 (0.57, 0.52–0.61)	3102 (1.01, 0.98–1.05)	1844 (1.20, 1.14–1.25)	1258 (0.82, 0.78–0.87)	4928 (0.87, 0.85–0.90)	2952 (1.03 0.99–1.07)	1976 (0.71, 0.68–0.74)

^a^ ASIRs, 95% CI and ASIRs are age‐standardized incidence rates to the new WHO standard population (per 100 000 person‐year).

The percentage of carcinomas (*n* = 2835, ASIR = 0.51), unspecified malignancies (*n* = 934, ASIR = 0.17), lymphomas (*n* = 704, IR = 0.11), sarcomas (*n* = 228, ASIR = 0.04), and neuroendocrine cancers (*n* = 214, ASIR = 0.04) accounted for approximately 57.5%, 19.0%, 14.3%, 4.6%, and 4.3% in all cases of SI cancers, respectively. Eighty‐one percentage of all kind of carcinomas were adenocarcinomas, not otherwise specified (NOS) (*n* = 2306, ASIR = 0.42). According to this study results, 52% of neuroendocrine cancers were carcinoid, NOS (*n* = 111, ASIR = 0.02); 67% of sarcomas were gastrointestinal stromal tumors (GIST) (n = 153, ASIR = 0.03). Besides, among all lymphoma cases, 37% were non‐Hodgkin lymphoma (NHL) and lymphoma‐NOS (*n* = 260, ASIR = 0.04) (Table 2 and Table S3).

**TABLE 2:** The frequency, ASIR^a^ (per 100 000 person‐year), SRR^b^, and AAPC^c^ of patients with small intestine cancers based on the main groups of the ICD‐O‐3, Iran, 2005–2015

ICD‐O‐3 group	No. of patients (ASIR)			SRR (95% CI)	ASIR (95% CI)		SRR (95% CI)	AAPC (95% CI)
	Total	Male	Female	Male to female	2005–2010	2010–2015	2010–2015 to 2005–2010	
Total	4928 (0.87)	2952 (1.03)	1976 (0.71)	1.45 (1.37–1.54)	0.70 (0.67–0.73)	1.01 (0.98–1.05)	1.44 (1.36–1.53)	9.6^d^ (5.7–13.7)
Carcinomas	2835 (0.51)	1619 (0.58)	1149 (0.42)	1.37 (1.27–1.48)	0.47 (0.45–0.50)	0.55 (0.52–0.57)	1.15 (1.07–1.24)	7.2^d^ (1.9–12.8)
Neuroendocrine cancers	214 (0.04)	111 (0.04)	102 (0.04)	1.11 (0.85–1.46)	0.02 (0.02–0.03)	0.05 (0.04–0.06)	2.19 (1.65–2.92)	17.8^d^ (5.8–31.2)
Sarcomas	228 (0.04)	111 (0.04)	114 (0.04)	0.94 (0.72–1.23)	0.03 (0.03–0.04)	0.04 (0.04–0.05)	1.25 (0.96–1.63)	3.6 (−11.9–21.9)
Lymphomas	704 (0.11)	440 (0.14)	238 (0.07)	1.85 (1.57–2.17)	0.12 (0.11–0.13)	0.10 (0.09–0.11)	0.81 (0.70–0.95)	−1.4 (−6.5–4.0)
Other specified malignancies	13 (<0.01)	14 (<0.01)	6 (<0.01)	2.56 (0.73–8.95)	<0.01	<0.01	—	—
Unspecified malignancies	934 (0.17)	557^d^ (0.20)	370 (0.14)	1.47 (1.29–1.68)	0.04 (0.04–0.05)	0.27 (0.25–0.29)	6.09 (5.17–7.17)	60.9^d^ (27.5–103.1)

^a^ Age‐standardized incidence rate to the new WHO standard population.

^b^ Standardized rate ratio.

^c^ Changes in trends among each line segment and overall lines were calculated using annual percent change (APC) and average annual percent change (AAPC), However, only AAPC was reported.

^d^ AAPC is significantly different from zero at the level of 0.05.

**FIGURE 2:**
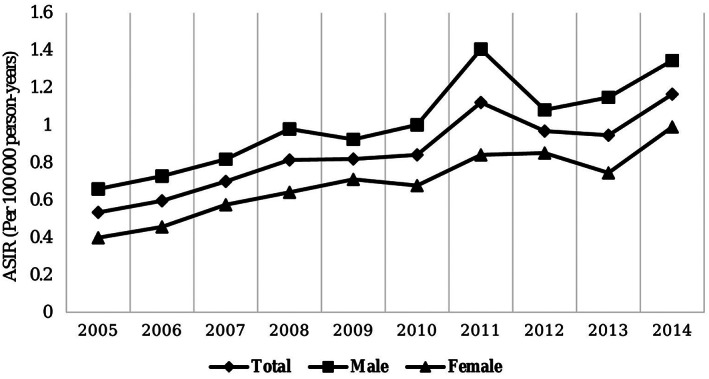
The ASIR (per 100 000 person‐year) of small intestine cancer, Iran, 2005–2015. ASIR, age‐standardized incidence rates

In the case of carcinomas and lymphomas, the ASIR in males were 0.58 and 0.14 respectively, per 100 000 person‐years, being 0.16 and 0.07 higher than females, respectively. However, the ASIR in neuroendocrine cancers and sarcomas were equal in males and females. In total, the ASIR experienced a significant increase during 2010–2015 in comparison to that of 2005–2010 (SRR = 1.44, 95% CI: 1.36–1.53). Over the course of this study, this increasing trend was observed at 9.6% (95% CI: 5.70–13.70) per year from 2005 to 2015. The upward trend of carcinomas and neuroendocrine cancers was illustrated at 7.20% and 17.80% per year during the course of the study, respectively. On the flip side, this measure in sarcomas and lymphomas similar to other major histological subtypes did not fluctuate significantly (Table 2). The ASIR of SI cancer had a rising trend despite some fluctuations (Figure 2).

Carcinomas' frequency was about seven times higher in the duodenum than in the jejunum and ileum. Moreover, the incidence of lymphoma and neuroendocrine cancers was higher in the duodenum than in the jejunum. The ASIR of carcinomas and lymphomas in the jejunum was significantly lower than the ASIR of this histology subtype in the duodenum. Similar results were observed for carcinomas and neuroendocrine cancers in the ileum compared to the duodenum. The ASIR of neuroendocrine cancer and sarcomas in the duodenum, jejunum, and ileum were not higher than 0.02 per 100 000 person‐years (Table 3).

**TABLE 3:** ASIR^a^ (per 100 000 person‐year) and SRR^b^ of small intestinal cancer according to subsite, Iran, 2005–2015

Subsite	Carcinomas			Neuroendocrine cancers			Sarcomas			Lymphomas		
Index	No.	ASIR	SRR (95% CI)	No.	ASIR	SRR (95% CI)	No.	ASIR	SRR (95% CI)	No.	ASIR	SRR (95% CI)
Duodenum	1221	0.23	(Reference)	67	0.01	(Reference)	21	<0.01	(Reference)	105	0.02	(Reference)
Jejunum	137	0.02	0.10 (0.09–0.12)	12	<0.01	—	31	0.01	—	49	0.01	0.44 (0.31–0.61)
Ileum	194	0.03	0.15 (0.13–0.17)	46	0.01	0.66 (0.45–0.96)	22	<0.01	—	122	0.02	1.12 (0.86–1.46)
Meckel diverticulum	4	<0.01	—	2	<0.01	—	1	<0.01	—	0	—	—
Overlapping lesion of SI	18	<0.01	—	0	—	—	1	<0.01	—	4	<0.01	—
Small intestine, NOS	1261	0.23	1.00 (0.93–1.09)	87	0.02	1.31 (0.95–1.81)	152	0.03	—	424	0.06	3.77 (3.09–4.61)

Abbreviations: NOS, not otherwise specified; SI, small intestine.

^a^ Age‐standardized incidence rate to the new WHO standard population.

^b^ Standardized rate ratio.

**FIGURE 3:**
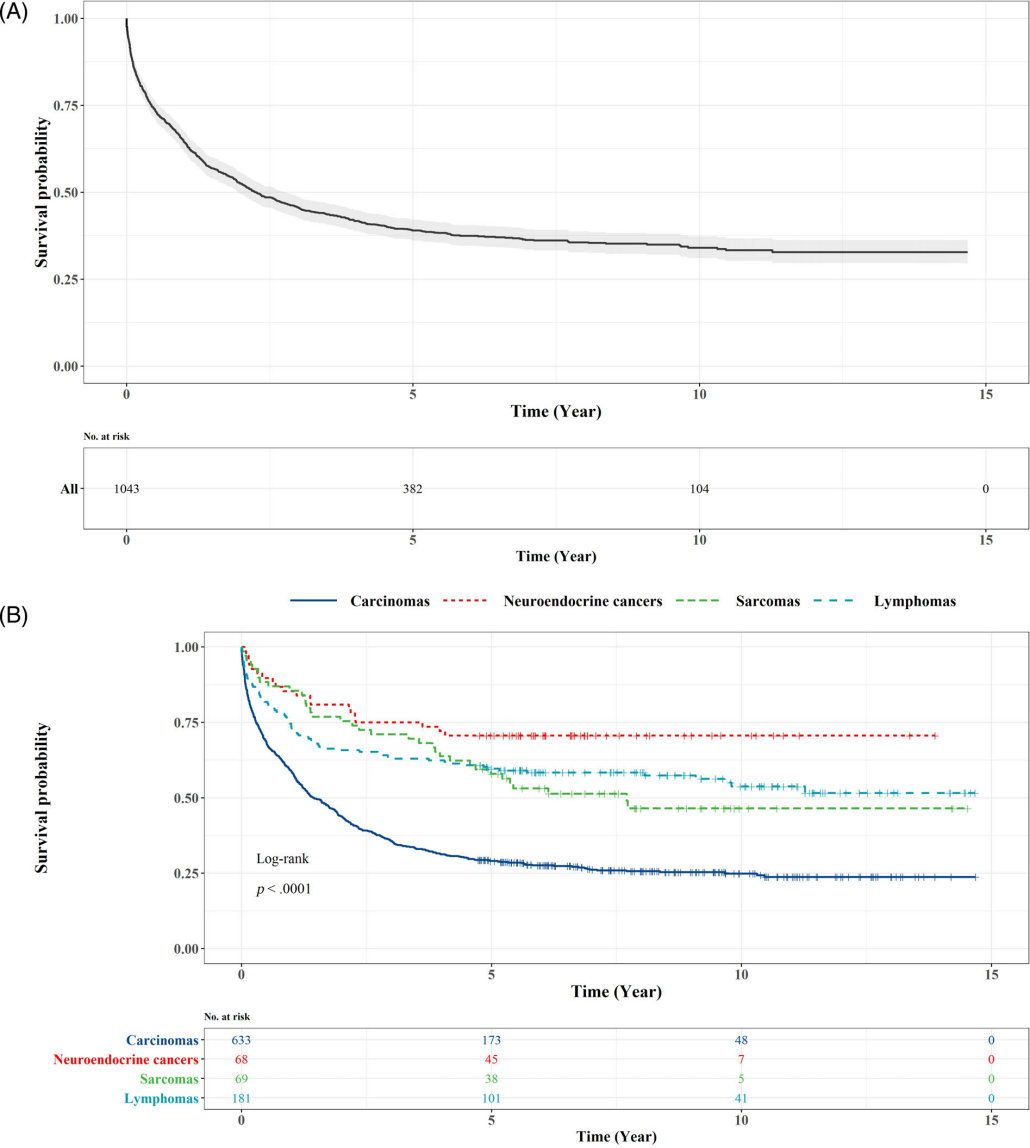
(A) The Kaplan–Meier survival curve of small intestine cancer, Iran, 2005–2015; (B) The Kaplan–Meier survival curve of small intestine cancer by histology types, Iran, 2005–2015

The overall 1‐ and 5‐year survival rate for all SI cancer were 62.4% and 35.3%, respectively. Among histological subtypes, the survival rates of patients with lymphomas, sarcomas, and neuroendocrine were moderate (more than 50.0%); however, the survival rate of carcinomas was the lowest (55.6 at 1 year and 24.1 at 5 years). Overall survival rate was significantly affected by gender (log‐rank test: *p*‐value <.05), while carcinomas patients had lower survival in males compared to females (log‐rank *p*‐values <.05). But other histology subtypes did not have significant different survival rates among males and females. The survival rate of carcinomas, sarcomas, and lymphomas patients who were younger than 60 was markedly higher than older patients. The 5‐year survival rate was significantly improved in lymphomas over the second 5 years of the study period compared to the first one (log‐rank test: *p*‐value <.05). The 5‐year survival rate of SI cancer located in the duodenum had the smallest percentage among carcinomas (21.0%) and sarcomas (25.0%). Moreover, the lowest survival rates of SI cancer occurred in the jejunum (35.0%) and ileum (39.5%) were carcinomas (Table 4). The Kaplan–Meier survival curve showed cumulative survival of patients about 0.3 after 11 years from the start of the study and remained stable until the end of the study (Figure 3A). In addition, the cumulative survival of patients revealed significant differences between different histology types (Figure 3B).

**TABLE 4:** Absolute survival rates of small intestinal cancer according to gender, age, calendar year, and subsite, Iran, 2005–2015

Characteristic	Carcinomas				Neuroendocrine cancers				Sarcomas				Lymphomas				Total^a^			
	No.	1 year (%)	5 years (%)	Sig.	No.	1 year (%)	5 years (%)	Sig.	No.	1 year (%)	5 years (%)	Sig.	No.	1 year (%)	5 years (%)	sig.	No.	1 year (%)	5 years (%)	Sig.
Total	592	55.6	24.1	—	66	84.8	69.7	—	69	85.5	57.9	—	168	70.8	56.5	—	983	62.4	35.3	—
Gender				0.106				0.086				0.773				0.168				0.025
Male	235	52.4	21.5		37	75.9	58.6		39	90.0	53.3		51	65.8	54.7		396	68.4	39.9	
Female	357	60.9	28.1		29	91.9	78.4		30	82.1	61.5		117	82.4	60.7		587	58.3	32.2	
Age (years)				<0.001				0.663				<0.001				0.003				<0.001
<60	253	66.8	34.7		37	86.5	73.0		43	90.7	69.8		123	77.2	63.4		498	72.1	47.6	
60+	339	47.5	16.2		29	82.8	65.5		26	76.9	38.5		45	53.3	37.8		485	52.4	22.7	
Calendar year				0.355				0.515				0.835				0.008				0.045
2005–2010	152	48.7	25.7		11	81.8	63.6		16	81.3	62.5		54	77.8	68.5		244	59.0	39.3	
2010–2015	440	58.2	23.6		55	85.5	70.9		53	86.8	56.5		114	67.5	50.8		739	63.5	33.9	
Subsite				0.145				0.516				0.118				0.327				<0.001
Duodenum	319	52.7	21.0		22	81.8	68.2		8	87.5	25.0		36	77.8	60.2		408	57.4	28.4	
Jejunum	31	61.3	35.5		4	—	—		7	85.7	—		12	66.7	58.3		59	71.2	50.8	
Ileum	38	73.7	39.5		17	88.2	76.5		8	87.5	50.0		31	77.4	58.1		97	80.4	54.6	
Meckel diverticulum	1	—	—		0	—	—		1	—	—		0	—	—		2	—	—	
Overlapping lesion of small intestine	9	66.7	33.3		0	—	—		0	—	—		0	—	—		9	66.7	33.3	
Small intestine, NOS	194	56.2	24.2		23	82.6	60.1		45	82.2	59.9		89	66.3	49.4		408	61.8	35.2	

Abbreviation: NOS, not otherwise specified.

^a^ The unspecified and other specified groups were considered in the total column.

## 4 DISCUSSION

The current study was the first population‐based study in Iran with the aim of investigating the incidence and absolute survival rate of SI cancer in different categories of sex, age, histology, and topography subtypes. Our findings revealed that the median age at diagnosis for both men and women was 61 years. Furthermore, the most frequent cases of SI cancer were found among patients aged 50–79. In addition, the cumulative incidence rate for those who are under 50 was less than one per 100 000 person‐years; however, the highest incidence rate was seen in those who were older than 75 with more than five per 100 000 person‐years. Overall, ASIR demonstrated an upward trend among both genders over the course of the study. Furthermore, the ASIR in the second 5 years of the study was approximately 45% higher than that of the first 5 years. The highest incidence rates in histology subtypes and subsites were reported in carcinomas and duodenum, respectively. Survival analysis in this study was performed using the cause‐specific survival method as an alternative to the relative‐survival method. Accordingly, the overall survival rate of the patients was estimated at about 30%, influenced by sex, age, and location of the cancer. Carcinomas had the lowest 5‐year survival rate among the four main histology subtypes. Moreover, patients with tumors located in the jejunum and ileum experienced the highest survival rates, whilst the lowest survival rates were reported for patients with the cancer located in the duodenum.

This study's findings indicated that the ASIR of SI cancer was approximately 1 per 100 000 person‐years in Iran, which was closely equal to the latest reported incidence rates in the world.^16^ Although SI cancer has been reported very rarely in the world, the United States and the United Kingdom have reported accurate ASIRs of 2.4 and 2.8 per 100 000 person‐years, respectively.^17^, ^18^, ^19^, ^20^, ^21^ It should be noted that various standard populations have been used in different studies to calculate the ASIR of SI cancer. In some studies conducted in the United States and Europe, the 2000 US standard population and 2013 European standard population were used separately. However, the new WHO standard population was used in the present study. Comparisons of ASIR based on different standard populations are inappropriate.^22^ The low incidence of small bowel tumors may be related to its minimal clinical symptoms. Most of these tumors are either asymptomatic for a long time or show nonspecific symptoms such as dull, abdominal pain, nausea, and vomiting. As the tumor grows in the SI, the disease risk progress increases, too; however, distinguishing between benign and malignant through clinical signs proves to be impossible.^23^, ^24^

The age distribution of SI cancer in Iran was quite similar to that of the United States and the United Kingdom. Based on the results, most of the new SI cancer cases in the United States and the United Kingdom occurred between the age of 55 and 84 years, with 73% and 72% of all cases respectively. Moreover, the age‐specific incidence rate of the cancer in these countries was higher than one per 100 000 person‐years for patients older than 45, and the peak ASIR of the cancer was observed in the age group of 80–84.^17^, ^18^, ^19^, ^20^, ^21^ This study results were consistent with the above‐mentioned findings, as per 76% of all new cases were in the age group of 55–84. Additionally, the incidence rate of SI cancer among Iranian patients older than 45 years was the same as the reported rate for the US and the UK patients in this age group. Furthermore, our findings showed a peak of ASIR in the 80–84 age group. The age might be a risk factor for SI cancer because the incidence rate of SI cancer has been on the rise from about the age of 40 onward until 84. In a study carried out by Hearle et al. in 2006, the risk of developing SI cancer increased rapidly from the age of 50 onward. Therefore, age could be considered as a risk factor for SI cancer.^25^

Few publications have reported on time trends in SI cancer incidence. In the United States, the incidence rate of this cancer has been increased by 2.2% per year in recent decades.^26^ However, in the United Kingdom, this trend has experienced a higher rate compared to the United States, with almost 5.6% in the last decade.^18^, ^19^, ^20^, ^21^ Recently, a study was conducted by Somi et al. in 2019 in the East Azerbaijan (a province of Iran) and the ASIR trend of SI cancer was calculated using AAPC within the years 2004 and 2015. According to their results, the cancer incidence rate in this province was increased at 7.6% from 2004–2015.^27^ These findings might indicate that the ASIR trend is escalating across the world. In our study, the ASIR trend of the SI cancer was also evaluated with the help of AAPC and SRR measures. Our findings showed that the ASIR trend was rising at 10% per year from 2005 to 2015. In addition, in the second 5‐year study, there was a significant increase in the ASIR compared to the first 5‐year study. There are various possible reasons for the increase in ASIR, especially in Iran. The increasing trend mentioned in this study could be due to the growth of the elderly population in Iran and the increased exposure to known risk factors. Improving the cancer registry process during the years of study in Iran may have increased reporting rates and completeness of registration.

Our findings showed that carcinomas and lymphomas constituted about 60% and 15% of SI cancers during 2005 and 2015, respectively. Besides, neuroendocrine cancers and sarcomas comprised less than 10% of all cases. As a result, carcinomas and lymphomas had the highest incidence rate, whilst the lowest incidence rate was reported for neuroendocrine cancer in Iran. However, Qubaiah et al.'s study demonstrated that neuroendocrine cancers and carcinomas were the most common histology subtypes, with 39% and 31% of all cases respectively, and lymphomas and sarcomas accounted for about 30% of new cancer cases during 1992–2006. According to their results, the incidence of neuroendocrine cancers was higher than the other histology subtypes followed by carcinomas. This difference in the results of the two studies might attribute to the difference between the two study periods. The present study was carried out from 2005 to 2015 while Qubiah et al.'s study was performed from 1992 to 2006. Their findings threw light on the fact that neuroendocrine cancer ASIRs rose notably across both genders over the course of study while the growth of cancer ASIR has been lower in other histology subtypes.^6^

In studies that were carried out within the United States as for the data from the Surveillance, Epidemiology and End Results (SEER) during 1973–2000 and 1992–2006, the ASIR of carcinomas experienced a slight rise in both of these periods from 0.72 to 1.45 in men and 0.50 to 1.00 in women per 100 000 person‐years.^6^, ^28^ According to ICD‐O3 classification, one of the main classes of carcinomas was adenocarcinomas.^29^ Approximately, 30%–40% of cancers observed in the SI were adenocarcinomas,^2^, ^30^ more common in men than women.^31^, ^32^, ^33^ Furthermore, the adenocarcinomas tumors were mainly located in the duodenum (50%–60%) and Jejunum/Ileum (25%–45%). In general, with respect to the latest epidemiological studies as for SI, the ASIR of adenocarcinoma was between 0.6 and 0.85 per 100 000 person‐years whereas adenocarcinomas located in duodenum, jejunum, and ileum had the ASIR of 0.3, 0.12, 0.09 per 100 000 person‐years, respectively.^2^, ^6^, ^30^, ^31^, ^34^ The ASIR trend of carcinomas in our study was almost the same as the mentioned ASIR trend in SEER programs while having different values. Besides, the findings of the present study showed that the ASIR of carcinomas was rising significantly in Iran; however, the value of ASIRs was approximately half of that reported rates in the above‐mentioned studies. Moreover, in the current study, adenocarcinomas accounted for about 50% of all SI cancers, and their incidence was slightly lower than that of the SEER program reports. Results of this study also showed that the ASIR of adenocarcinoma in the duodenum was higher in the jejunum and ileum, but this rate was generally lower compared to other studies. This can be ascribed to improved diagnosis resulting in a reduction of misclassification of adenocarcinoma tumors as adenocarcinoma of unknown primary in high‐income countries compared to low‐income countries such as Iran. In our study, 45% of adenocarcinomas subsites were unknown, reflecting the need for improving protocols for diagnostic and prognostic tests.

The current study showed that less than 5% of SI cancer cases were neuroendocrine cancers with ASIR less than 0.1 per 100 000 person‐years. Moreover, the ASIR of this histology subtype in the second 5 years of the study had a significant growth compared to the first 5 years and the AAPC of this cancer experienced a significant upward trend. This study's findings illustrated that the two main subgroups of neuroendocrine cancers were carcinoid and large cell tumors made up more than 90% of all cases. In addition, more than half of neuroendocrine cancers occurred in the duodenum and ileum. The results of this study were inconsistent with the other findings regarding neuroendocrine cancers. The standardized incidence of neuroendocrine SI cancer in the United States from 1992 to 2006 was 0.83 per 100 000 person‐years, and almost all cases of neuroendocrine cancer were carcinoid. According to the results of this study, the ASIR of carcinoid and large cell was 0.75 and 0.06 per 100 000 person‐years, respectively.^6^, ^35^ Furthermore, most of the neuroendocrine cancers were located in the ileum whereas the rare cases were found in the duodenum.^2^, ^36^ In another study conducted by Legué et al. in 2016, the percentage of histology subtypes of SI cancer was obtained and neuroendocrine cancer was the second most frequent among them.^34^ The observation of higher incidences of neuroendocrine carcinomas in recent years is probably due to improved diagnostic procedures in high‐income countries.^37^ Nearly a thousand SI cancers were unspecified in our study some of which may be classified as neuroendocrine cancer with regard to improving diagnostic tools at health centers in Iran. Furthermore, a large number of unspecified cases were found in neuroendocrine cancers. Small ileum cases might be ascribed to the lack of diagnosis and identification methods for these histology subtypes.

Based on a few studies, sarcomas were regarded as one of the rarest histology subtypes of SI about which there were few epidemiological reports. Moreover, sarcomas accounted for only 10%–15% of cancer cases in the SI.^28^, ^30^, ^35^, ^38^ In three published papers by Weiss et al. in 1987, Haselkorn et al. in 2005, and Schottenfeld et al. in 2013, the ASIR of sarcomas were 0.12, 0.17, and 0.18 per 100 000 person‐years, respectively.^28^, ^30^, ^36^ Recent studies showed that the most common type of sarcomas was GISTs occurred in SI. In addition, approximately 35% of GISTs were developed in SI and about 90% of SI sarcomas were GISTs.^39^, ^40^ On average, 25% of SI sarcomas were found in the jejunum, 19% in the ileum, and 16% in the duodenum.^28^, ^30^, ^38^ Our findings were almost consistent with the above‐mentioned results. However, sarcomas were more common than neuroendocrine cancers, with ASIR less than 0.05 per 100 000 person‐years. Moreover, a large percentage of SI sarcomas in our study were GIST, which was more common in jejunum and ileum, respectively.

The current study revealed that lymphomas were the second most common histology subtypes of SI cancer after sarcomas in Iran. A large number of this histology subtype were large B‐cells and NHL/lymphoma, NOS. Besides, lymphomas were often observed in the ileum and duodenum; however, the location of more than 60% of lymphomas were unknown. The results of other studies were almost in line with this study's findings. In Qubaiah et al.'s study, lymphomas were the third most common type of SI cancer while most cases of lymphomas were composed of large B‐Cell. Their findings showed that about 45% of lymphomas were located in the ileum and duodenum and about 40% were unspecified.^6^ In a study by Haselkorn et al., ileum, and then jejunum were reported as the locations where lymphomas occurred more than other subsites.^28^

Various studies have shown that the 5‐year survival rate of SI cancer was between 40% and 68%. In almost all studies, the survival rate of SI cancer was affected by age and sex. Howe et al. showed that tumor size, histologic type, stage, grade, and surgical status were the significant risk factors for survival of SI cancer. In a study by Bilimoria et al., the survival of patients in four histology subtypes was investigated and only the survival of adenocarcinoma was influenced by subsites. According to their studies, hazard ratio of duodenum and ileum was higher than the jejunum. In the study by Qubaiah et al., a descriptive analysis of survival rates in terms of the ICDO‐3 histology subtypes was performed in which the 5‐year survival rates of carcinomas and neuroendocrine cancers were 28% and 81%, respectively. According to their study, the duodenum had the lowest survival rate among all histology subtypes.^6^, ^38^, ^41^, ^42^ In this study, the 5‐year survival rate of SI cancer was relatively less compared to the above‐mentioned studies. One reason could be due to nonspecific symptoms and infrequent occurrence of SI cancer, often leading to a delay in diagnosis and consequently poor prognosis. In addition, poor diagnostic tools in deprived areas of Iran could lead to delays in the early detection of this cancer; therefore, as a result, the patient might have less chance of surviving. This study's findings on survival rates in histologic subtypes were consistent with other recent studies. Accordingly, carcinomas had the lowest 5‐year survival rate while neuroendocrine cancers had the highest one. Besides, our study was consistent with the findings of the above‐mentioned studies about the survival rate of the duodenum. Although many of the risk factors were not assessed in our study, some factors including age, sex, and histology subsite were identified as significant risk factors for SI cancer survival.

None of the studies in Iran used the full‐coverage cancer registry. Population‐based cancer registration in Iran is relatively incomplete, making it difficult to generalize and compare results with other comprehensive registries. The limited number of cancer research centers have access to Social Security Insurance data in Iran, including financial insurance services of registered cancer patients, resulting in significant elimination of the incomplete data.^43^ However, few risk factors were taken into account in the case of Iranian cancer registries which makes it impossible to examine the relationship between incidence rate (or its trend) and important risk factors.^44^ Improving registration of SI cancers requires faster and more accurate diagnosis alongside with more complete registration. On the one hand, the rapid identification and correct diagnosis of cancer should be available and affordable in cancer centers for everybody. On the other hand, software and hardware resources should be promoted to register cancer data precisely and completely. In addition, individuals and staffs who are going to record data should be well‐informed and fully aware of the importance of accurate data registration.

Our study's bright side was the application of the first and most comprehensive data about SI cancer, diagnosed in a population‐based setting in Iran based on the ICD‐O3 classification. However, this study also faced with some limitations. A major limitation is that the completeness of the INCR is not known, which can cause bias in the results and underestimation of incidence. Furthermore, the incidence of histology subtypes at different age groups was not investigated by gender. In addition, some information in our dataset were not listed, including tumor size, stage, grade, and type of treatment.

In conclusion, the pattern of SI cancer in this study was slightly different from other countries. The total incidence rate of SI cancer in Iran was lower than high‐income countries. Furthermore, the neuroendocrine cancer was a rare malignancy in Iran; however, this histology subtype was one of the most common types of SI cancer in other countries. Moreover, the poor survival rate revealed need of early detection and early diagnosis of SI cancer in Iran.

## CONFLICT OF INTEREST

The authors have stated explicitly that there are no conflicts of interest in connection with this article.

## AUTHOR CONTRIBUTION

All authors had full access to the data in the study and take responsibility for the integrity of the data and the accuracy of the data analysis. *Conceptualization*, M.E.A., S.K.H.; *Methodology*, A.M.L., S.K.H.; *Validation*, A.M.L., E.Z.; *Investigation*, A.M.L., S.K.H., E.Z.; *Writing ‐ Original Draft*, A.M.L., E.Z.; *Writing ‐ Review & Editing*, M..E.A., S.K.H.; *Supervision*, M.E.A.; *Project Administration and Funding Acquisition*, S.K.H.

## ETHICAL STATEMENT

The current study was approved by the Ethics Committee of Shahid Beheshti University of Medical Sciences (IR.SBMU.CRC.REC.1399.004).

## Supporting information


**Table S1.** Percentage of data extraction.
**Table S2.** Five‐year annual frequency (age‐specific incidence rate [per 100 000 person‐year], 95% CI) for small intestine cancer, Iran, 2005–2015.
**Table S3.** Frequency, ASIR (per 100 000 person‐year), SRR, and AAPC of patients with small intestine cancer based on ICD‐O‐3, Iran, 2005–2015.

## Data Availability

The data that supports the findings of this study are available from the corresponding author upon reasonable request.
