# Supplementary material for: Epidemiology of small intestine cancer in Iran
Source: Cancer Rep (Hoboken). 2021 Nov 25;5(10):e1593. doi: 10.1002/cnr2.1593 (PMC9575508; doi:10.1002/cnr2.1593)
Supplement: Supplementary file 1 — Table S1. Percentage of data extraction. Table S2. Five‐year annual frequency (age‐specific incidence rate [per 100 000 person‐year], 95% CI) for small intestine cancer, Iran, 2005–2015. Table S3. Frequency, ASIR (per 100 000 person‐year), SRR, and AAPC of patients with small intestine cancer based on ICD‐O‐3, Iran, 2005–2015. [file CNR2-5-e1593-s001.docx]

**Supplementary**

Table S1. Percentage of data extraction

| **Year** | **Before duplicate extraction** | **After duplicated extraction** | **Percentage** |
| --- | --- | --- | --- |
| 2005* | 262 | 262 | 0.00 |
| 2006 | 308 | 306 | 0.65 |
| 2007 | 376 | 369 | 1.86 |
| 2008 | 434 | 433 | 0.23 |
| 2009 | 458 | 456 | 0.44 |
| 2010 | 487 | 487 | 0.00 |
| 2011 | 698 | 667 | 4.44 |
| 2012 | 632 | 598 | 5.38 |
| 2013 | 647 | 603 | 6.80 |
| 2014 | 754 | 747 | 0.93 |
| Total | 5056 | 4928 | 2.53 |

* Note: The study started in March 2005 and continued until March 2015. The Solar Hijri year (Iranian Hijri calendar) begins about 21 March of each Gregorian year and ends about 20 March of the next year. Therefore, the study ended in March 2015.

Table S2. Five-year annual frequency (age-specific incidence rate (per 100,000 person-year), 95% CI) for small intestine cancer, Iran, 2005-2015.

| **Age Group** | **2005-2010** | | | **2010-2015** | | | **2005-2015** | | |
| --- | --- | --- | --- | --- | --- | --- | --- | --- | --- |
|  | **Total** | **Male** | **Female** | **Total** | **Male** | **Female** | **Total** | **Male** | **Female** |
| **0-4** | 17 (0.06, 0.03-0.09) | 12 (0.08, 0.04-0.13) | 5 (0.04, 0.00-0.07) | 40 (0.13, 0.09-0.16) | 24 (0.15, 0.09-0.20) | 16 (0.10, 0.05-0.15) | 57 (0.09, 0.07-0.12) | 36 (0.12, 0.08-0.16) | 21 (0.07, 0.04-0.10) |
| **5-9** | 25 (0.09, 0.05-0.13) | 18 (0.13, 0.07-0.19) | 7 (0.05, 0.01-0.09) | 17 (0.06, 0.03-0.09) | 13 (0.09, 0.04-0.13) | 4 (0.03, 0.00-0.06) | 42 (0.07, 0.05-0.10) | 31 (0.11, 0.07-0.14) | 11 (0.04, 0.02-0.06) |
| **10-14** | 15 (0.05, 0.02-0.07) | 10 (0.06, 0.02-0.10) | 5 (0.03, 0.00-0.06) | 13 (0.05, 0.02-0.07) | 11 (0.08, 0.03-0.12) | 2 (0.01, -0.01-0.03) | 28 (0.05, 0.03-0.06) | 21 (0.07, 0.04-0.10) | 7 (0.02, 0.01-0.04) |
| **15-19** | 27 (0.07, 0.04-0.09) | 19 (0.09, 0.05-0.13) | 8 (0.04, 0.01-0.07) | 23 (0.07, 0.04-0.10) | 15 (0.09, 0.05-0.14) | 8 (0.05, 0.02-0.09) | 50 (0.07, 0.05-0.09) | 34 (0.09, 0.06-0.12) | 16 (0.04, 0.02-0.07) |
| **20-24** | 38 (0.09, 0.06-0.11) | 27 (0.12, 0.08-0.17) | 11 (0.05, 0.02-0.08) | 54 (0.14, 0.10-0.17) | 39 (0.20, 0.13-0.26) | 15 (0.08, 0.04-0.11) | 92 (0.11, 0.09-0.13) | 66 (0.16, 0.12-0.19) | 26 (0.06, 0.04-0.09) |
| **25-29** | 65 (0.17, 0.13-0.22) | 32 (0.17, 0.11-0.23) | 33 (0.18, 0.12-0.24) | 52 (0.12, 0.09-0.16) | 28 (0.13, 0.08-0.18) | 24 (0.11, 0.07-0.16) | 117 (0.15, 0.12-0.17) | 60 (0.15, 0.11-0.19) | 57 (0.14, 0.11-0.18) |
| **30-34** | 71 (0.24, 0.19-0.30) | 44 (0.30, 0.21-0.38) | 27 (0.19, 0.12-0.26) | 91 (0.25, 0.20-0.30) | 62 (0.34, 0.25-0.42) | 29 (0.16, 0.10-0.22) | 162 (0.25, 0.21-0.29) | 106 (0.32, 0.26-0.38) | 56 (0.17, 0.13-0.22) |
| **35-39** | 77 (0.31, 0.24-0.37) | 47 (0.36, 0.26-0.47) | 30 (0.24, 0.16-0.33) | 96 (0.33, 0.26-0.39) | 58 (0.39, 0.29-0.49) | 38 (0.26, 0.18-0.35) | 173 (0.32, 0.27-0.36) | 105 (0.38, 0.30-0.45) | 68 (0.25, 0.19-0.31) |
| **40-44** | 97 (0.46, 0.37-0.55) | 58 (0.54, 0.40-0.68) | 39 (0.37, 0.26-0.49) | 164 (0.65, 0.55-0.75) | 100 (0.79, 0.63-0.94) | 64 (0.52, 0.39-0.65) | 261 (0.56, 0.50-0.63) | 158 (0.67, 0.57-0.78) | 103 (0.45, 0.36-0.54) |
| **45-49** | 145 (0.80, 0.67-0.93) | 84 (0.91, 0.72-1.11) | 61 (0.68, 0.51-0.86) | 223 (1.06, 0.92-1.20) | 127 (1.20, 0.99-1.41) | 96 (0.92, 0.74-1.11) | 368 (0.94, 0.85-1.04) | 211 (1.07, 0.92-1.21) | 157 (0.81, 0.69-0.94) |
| **50-54** | 191 (1.32, 1.13-1.50) | 122 (1.67, 1.38-1.97) | 69 (0.96, 0.73-1.18) | 316 (1.76, 1.57-1.96) | 176 (1.96, 1.67-2.25) | 140 (1.57, 1.31-1.82) | 507 (1.56, 1.43-1.70) | 298 (1.83, 1.62-2.04) | 209 (1.29, 1.12-1.47) |
| **55-59** | 165 (1.62, 1.37-1.87) | 95 (1.90, 1.52-2.29) | 70 (1.35, 1.03-1.67) | 297 (2.13, 1.88-2.37) | 182 (2.63, 2.25-3.01) | 115 (1.63, 1.33-1.93) | 462 (1.91, 1.74-2.09) | 277 (2.32, 2.05-2.60) | 185 (1.51, 1.29-1.73) |
| **60-64** | 202 (2.62, 2.26-2.98) | 100 (2.65, 2.13-3.16) | 102 (2.60, 2.10-3.11) | 329 (3.30, 2.94-3.65) | 193 (4.04, 3.47-4.61) | 136 (2.62, 2.18-3.06) | 531 (3.00, 2.75-3.26) | 293 (3.42, 3.03-3.82) | 238 (2.61, 2.28-2.94) |
| **65-69** | 163 (2.66, 2.25-3.07) | 99 (3.16, 2.54-3.78) | 64 (2.14, 1.61-2.66) | 300 (4.22, 3.75-4.70) | 158 (4.65, 3.92-5.37) | 142 (3.84, 3.21-4.47) | 463 (3.50, 3.18-3.82) | 257 (3.93, 3.45-4.42) | 206 (3.08, 2.66-3.50) |
| **70-74** | 209 (3.73, 3.23-4.24) | 140 (4.74, 3.95-5.53) | 69 (2.61, 1.99-3.23) | 340 (6.00, 5.36-6.64) | 213 (7.54, 6.53-8.55) | 127 (4.47, 3.69-5.25) | 549 (4.87, 4.47-5.28) | 353 (6.11, 5.47-6.75) | 196 (3.57, 3.07-4.07) |
| **75-79** | 175 (4.76, 4.05-5.46) | 111 (5.66, 4.60-6.71) | 64 (3.73, 2.82-4.65) | 337 (7.51, 6.71-8.31) | 203 (8.70, 7.51-9.90) | 134 (6.22, 5.17-7.28) | 512 (6.27, 5.73-6.81) | 314 (7.31, 6.50-8.12) | 198 (5.12, 4.41-5.83) |
| **80-84** | 108 (5.09, 4.13-6.05) | 73 (6.69, 5.15-8.22) | 35 (3.40, 2.27-4.52) | 259 (8.72, 7.66-9.79) | 153 (10.14, 8.53-11.75) | 106 (7.26, 5.88-8.65) | 367 (7.21, 6.47-7.95) | 226 (8.69, 7.56-9.82) | 141 (5.66, 4.73-6.60) |
| **85+** | 36 (2.57, 1.73-3.41) | 17 (2.34, 1.23-3.46) | 19 (2.82, 1.55-4.08) | 151 (7.80, 6.56-9.04) | 89 (8.93, 7.08-10.79) | 62 (6.60, 4.95-8.24) | 187 (5.61, 4.80-6.41) | 106 (6.16, 4.99-7.33) | 81 (5.02, 3.92-6.11) |

Table S3. Frequency, ASIR (per 100,000 person-years), SRR and AAPC of patients with small intestine cancer based on ICD-O-3, Iran, 2005-2015.

| **ICD-O3 Group** | **No. (ASIR)** | | | **SRR (95% CI)** | **ASIR (95% CI)** | | **SRR (95% CI)** | **AAPC (95% CI)** |
| --- | --- | --- | --- | --- | --- | --- | --- | --- |
|  | **Total** | **Male** | **Female** | **Male**  **to**  **Female** | **2005-2010** | **2010-2015** | **2010-2015**  **to**  **2005-2010** |  |
| **Total** | **4928 (0.87)** | **2952 (1.03)** | **1976 (0.71)** | **1.45 (1.37-1.54)** | **0.70 (0.67-0.73)** | **1.01 (0.98-1.05)** | **1.44 (1.36-1.53)** | **9.6^*^ (5.7-13.7)** |
| **Carcinomas** | **2835 (0.51)** | **1619 (0.58)** | **1149 (0.42)** | **1.37 (1.27-1.48)** | **0.47 (0.45-0.50)** | **0.55 (0.52-0.57)** | **1.15 (1.07-1.24)** | **7.2* (1.9-12.8)** |
| Adenocarcinomas |  |  |  |  |  |  |  |  |
| NOS | 2306 (0.42) | 1371 (0.49) | 935 (0.34) | 1.43 (1.31-1.55) | 0.39 (0.37-0.42) | 0.44 (0.42-0.47) | 1.12 (1.03-1.22) | 6.7^*^ (1.3-12.5) |
| Mucin-producing/mucinous | 216 (0.04) | 133 (0.05) | 83 (0.03) | 1.57 (1.19-2.06) | 0.04 (0.03-0.05) | 0.04 (0.03-0.04) | 0.92 (0.70-1.20) | 2.3 (-4.7-9.9) |
| Arising in adenoma | 15 (<0.01) | 10 (<0.01) | 5 (<0.01) | 1.79 (0.62-5.19) | <0.01 | <0.01 | --- | --- |
| Other specified | 78 (0.01) | 50 (0.02) | 28 (0.01) | 1.80 (1.13-2.85) | 0.02 (0.01-0.02) | 0.01 (0.01-0.02) | 0.92 (0.58-1.44) | 10.1 (-19.3-50.2) |
| *Other Specified* | 27 (<0.01) | 13 (<0.01) | 14 (<0.01) | 0.96 (0.45-2.06) | <0.01 | <0.01 | --- | --- |
| NOS | 193 (0.03) | 109 (0.04) | 84 (0.03) | 1.23 (0.92-1.64) | 0.02 (0.02-0.03) | 0.05 (0.04-0.05) | 2.04 (1.51-2.76) | 15.2^*^ (2.2-29.9) |
| **Neuroendocrine cancers** | **214 (0.04)** | **111 (0.04)** | **102 (0.04)** | **1.11 (0.85-1.46)** | **0.02 (0.02-0.03)** | **0.05 (0.04-0.06)** | **2.19 (1.65-2.92)** | **17.8* (5.8-31.2)** |
| Differentiated (carcinoids) |  |  |  |  |  |  |  |  |
| Carcinoid, NOS | 111 (0.02) | 57 (0.02) | 54 (0.02) | 1.10 (0.75-1.61) | 0.02 (0.01-0.02) | 0.02 (0.02-0.03) | 1.26 (0.86-1.85) | 8.8 (-8.7–29.6) |
| Hormone-specific | 3 (<0.01) | 3 (<0.01) | --- | --- | <0.01 | <0.01 | --- |  |
| Undifferentiated |  |  |  |  |  |  |  |  |
| Large cell | 88 (0.02) | 45 (0.02) | 43 (0.02) | 1.04 (0.68-1.59) | 0.00 (0.00-0.01) | 0.03 (0.02-0.03) | 5.60 (3.31-9.45) | --- |
| Small cell | 4 (<0.01) | 4 (<0.01) | --- | --- | <0.01 | <0.01 | --- | --- |
| Atypical carcinoid | 6 (<0.01) | 3 (<0.01) | 3 (0.00) | 1.07 (0.21-5.61) | <0.01 | <0.01 | --- | --- |
| Mixed endocrine-exocrine | 2 (<0.01) | 1 (<0.01) | 1 (0.00) | 0.75 (0.04-12.53) | <0.01 | <0.01 | --- | --- |
| **Sarcomas** | **228 (0.04)** | **111 (0.04)** | **114 (0.04)** | **0.94 (0.72-1.23)** | **0.03 (0.03-0.04)** | **0.04 (0.04-0.05)** | **1.25 (0.96-1.63)** | **3.6 (-11.9-21.9)** |
| GIST | 153 (0.03) | 80 (0.03) | 73 (0.03) | 1.06 (0.77-1.46) | 0.02 (0.02-0.03) | 0.03 (0.02-0.03) | 1.17 (0.85-1.63) | 2.7 (-13.5-21.9) |
| Leiomyosarcoma | 34 (0.01) | 14 (<0.01) | 20 (0.01) | 0.69 (0.35-1.38) | <0.01 | 0.01 (0.00-0.01) | --- | 3.9 (-22.7-39.7) |
| Other specified | 6 (<0.01) | 4 (<0.01) | 2 (<0.01) | 1.77 (0.33-9.59) | <0.01 | <0.01 | --- | --- |
| Undifferentiated | 35 (0.01) | 16 (<0.01) | 19 (0.01) | 0.76 (0.38-1.50) | 0.01 (0.00-0.01) | 0.01 (0.00-0.01) | 1.24 (0.63-2.46) | 9.1 (-34.8-82.4) |
| **Lymphomas** | **704 (0.11)** | **440 (0.14)** | **238 (0.07)** | **1.85 (1.57-2.17)** | **0.12 (0.11-0.13)** | **0.10 (0.09-0.11)** | **0.81 (0.70-0.95)** | **-1.4 (-6.5-4.0)** |
| B-cell |  |  |  |  |  |  |  |  |
| Large cell | 234 (0.04) | 157 (0.05) | 77 (0.02) | 2.18 (1.65-2.87) | 0.04 (0.03-0.05) | 0.03 (0.03-0.04) | 0.78 (0.59-1.01) | -4.2 (-10.8-2.8) |
| Follicular | 3 (<0.01) | 3 (<0.01) | --- | --- | <0.01 | 0.00 (0.00-0.00) | 1.85 (0.18-19.08) | --- |
| MALT | 62 (0.01) | 39 (0.01) | 23 (0.01) | 1.72 (1.01-2.91) | 0.01 (0.01-0.02) | 0.01 (0.00-0.01) | 0.64 (0.38-1.08) | -5.7 (-23.3-15.8) |
| Diffuse | 21 (<0.01) | 16 (0.01) | 6 (<0.01) | 2.98 (1.17-7.64) | 0.01 (0.00-0.01) | <0.01 | 0.27 (0.10-0.74) | --- |
| Burkitt | 71 (0.01) | 50 (0.01) | 19 (0.01) | 2.31 (1.39-3.83) | 0.01 (0.01-0.01) | 0.01 (0.01-0.01) | 1.01 (0.63-1.62) | 1.1 (-14.4-19.5) |
| Other specified | 15 (<0.01) | 10 (<0.01) | 5 (<0.01) | 2.25 (0.76-6.69) | <0.01 | <0.01 | --- | --- |
| T-cell | 38 (0.01) | 26 (0.01) | 13 (<0.01) | 2.07 (1.06-4.03) | <0.01 | 0.01 (0.00-0.01) | --- | 7.0 (-44.2-105.2) |
| NHL and lymphoma NOS | 260 (0.04) | 155 (0.05) | 95 (0.03) | 1.53 (1.18-1.98) | 0.05 (0.04-0.05) | 0.04 (0.03-0.04) | 0.79 (0.61-1.02) | -1.1 (-16.7-17.5) |
| **Other specified malignancies** | **13 (<0.01)** | **14 (<0.01)** | **6 (<0.01)** | **2.56 (0.73-8.95)** | **<0.01** | **<0.01** | **---** | **---** |
| Melanoma | 6 (<0.01) | 4 (<0.01) | 1 (<0.01) | 4.50 (0.56-36.36) | <0.01 | <0.01 | --- | --- |
| Neuroepithelial and peripheralnerve sheath | 5 (<0.01) | 3 (<0.01) | 2 (<0.01) | 1.24 (0.20-7.58) | <0.01 | <0.01 | --- | --- |
| Myeloid sarcoma | 2 (<0.01) | 2 (<0.01) | 0 (<0.01) | --- | <0.01 | <0.01 | --- | --- |
| **Unspecified malignancies** | **934 (0.17)** | **557 (0.20)** | **370 (0.14)** | **1.47 (1.29-1.68)** | **0.04 (0.04-0.05)** | **0.27 (0.25-0.29)** | **6.09 (5.17-7.17)** | **60.9^*^ (27.5-103.1)** |

ASIR: Age-standardized incidence rates to the new WHO standard population, SRR: Standardized rate ratio, AAPC: average annual percent change.

* Significantly different from zero at the level of 0.05.
